# Genetic Background and Climatic Droplet Keratopathy Incidence in a Mapuche Population from Argentina

**DOI:** 10.1371/journal.pone.0074593

**Published:** 2013-09-05

**Authors:** Theodore G. Schurr, Matthew C. Dulik, Thamara A. Cafaro, María F. Suarez, Julio A. Urrets-Zavalia, Horacio M. Serra

**Affiliations:** 1 Department of Anthropology, University of Pennsylvania, Philadelphia, Pennsylvania, United States of America; 2 CIBICI (Centro de Investigaciones en Bioquímica Clínica e Inmunología), Faculty of Chemistry, National University of Córdoba, Córdoba, Argentina; 3 Department of Ophthalmology, Catholic University of Córdoba, Córdoba, Argentina; University of Birmingham, United Kingdom

## Abstract

**Purpose:**

To determine whether the incidence of and susceptibility to climatic droplet keratopathy (CDK), an acquired, often bilateral degenerative corneal disease, is influenced by the genetic background of the individuals who exhibit the disorder.

**Methods:**

To determine whether the disease expression was influenced by the genetic ancestry of CDK cases in native Mapuche of the northwest area of Patagonia in Argentina, we examined mitochondrial DNA and Y-chromosome variation in 53 unrelated individuals. Twenty-nine of them were part of the CDK (patient) population, while 24 were part of the control group. The analysis revealed the maternal and paternal lineages that were present in the two study groups.

**Results:**

This analysis demonstrated that nearly all persons had a Native American mtDNA background, whereas 50% of the CDK group and 37% of the control group had Native American paternal ancestry, respectively. There was no significant difference in the frequencies of mtDNA haplogroups between the CDK patient and control groups. Although the Y-chromosome data revealed differences in specific haplogroup frequencies between these two groups, there was no statistically significant relationship between individual paternal genetic backgrounds and the incidence or stage of disease.

**Conclusions:**

These results indicate a lack of correlation between genetic ancestry as represented by haploid genetic systems and the incidence of CDK in Mapuche populations. In addition, the mtDNA appears to play less of a role in CDK expression than for other complex diseases linked to bioenergetic processes. However, further analysis of the mtDNA genome sequence and other genes involved in corneal function may reveal the more precise role that mitochondria play in the expression of CDK.

## Introduction

Climatic droplet keratopathy (CDK) is an acquired, generally bilateral degenerative disease of the cornea characterized by its slowly progressive opacity, affecting predominantly males over 40 years old [1]. A clinically well-defined entity, CDK is characterized by the progressively hazy appearance of the cornea caused by the opalescence of its most anterior layers. It progresses through three stages of increasing severity [2,3]. In *Grade 1* of CDK, multiple and tightly confluent small, translucent subepithelial droplet-like deposits are observed in the peripheral cornea, close to the temporal and/or nasal limbus, leaving a prelimbal fringe of apparently normal cornea. At this initial stage, where these peripheral microdroplets are best seen biomicroscopically with back-scattered slit-illumination and high magnification, no compromise of visual acuity occurs. In *Grade 2*, this haziness extends over the central cornea in a horizontal band-shaped distribution, giving a tarnished appearance of the inferior two thirds of the cornea, blurring the details of the iris under the diseased areas. By this stage, visual acuity of the affected eye may be severely compromised. In *Grade 3*, clusters of confluent yellow and amber-like subepithelial droplets of different sizes are observed distributed throughout areas of microdroplet opalescence [2,3].

Cornea’s hypoesthesia has been observed in many CDK patients, being more severe in advanced stages of the disease [4]. This decrease of corneal sensitivity may lead to severe trophic changes that predispose to ulceration, spontaneous perforation and eye’s atrophy and irreversible blindness. Sequels of corneal perforations were more common in patients with CDK than in controls of similar age [5]. Occurrences of severe corneal ulcers complicated by bacterial infection, and their torpid evolution to perforation and endophthalmitis have also been reported in CDK [6,7].

In histopathological light microscope examination, globular deposits of different sizes may be observed under the corneal epithelium, within Bowman’s membrane and the anterior stroma. The coalescence and increased volume of these spherules or deposits may cause disruption of Bowman’s membrane and elevation and thinning of the corneal epithelium [1,8,9,10]. Electron microscopy has shown that globules are round, electron-dense and sharply demarcated structures, always surrounded by basement membrane material, and adjacent disorganized collagen fibrils [1,9]. The droplets generally lack positive staining for fat or calcium [1]. Although the exact nature and composition of droplets remain unclear, proteinaceous constituents have been found [8,9,10,11].

More recently, Kaji et al. [12] found immunoreactivity against advanced glycation end products (AGE) in cornea of CDK patients when compared with normal cornea and other corneal diseases such as bullous keratopathy or band keratopathy. This finding led them to postulate that pathogenesis of CDK might be an aggregation of AGE-modified proteins as the consequence of aging and ultraviolet radiation (UVR) exposure.

Using glycopeptide capture and iTRAQ, we sampled tears from CDK patients and controls, and identified a total of 43 unique N-glycoproteins, 19 of which have not previously been reported in tear fluid. The quantitative study in patients’ tears showed increased levels of four N-glycosylated proteins, including haptoglobin, polymeric immunoglobulin receptor, immunoglobulin J chain and an uncharacterized protein DKFZp686M08189, as well as a decrease in the N glycosylation level of lacritin [13]. More recently, we identified approximately 105 proteins in droplets of CDK specimens using proteomic analysis, a subset of them being confirmed by immunohistochemistry [14]. The most frequent pathway for which the proteins have been identified were cell junction, focal adhesion and regulation of cytoskeleton, in addition to energy metabolism associated proteins, suggesting that several of them could play a role in fibril or deposit formation [14].

CDK may be observed in an otherwise healthy eye. However, pinguecula, pterigum, cataract and pseudo exfoliation have been frequently observed among patients with CDK [3,15]. More recently, iris atrophy has also been noted in CDK patients [4].

Although the etiology of CDK is unknown, it is considered a multifactorial disease related to environmental factors. Intense constant winds, lack of shade, low humidity and UVR exposure in hot as well as cold arid climates are the more common environmental factors observed in areas with high prevalence of the disease. For these reasons, CDK is considered a rural and an outdoor labor disease, and very rarely affects urban individuals [3,4,16,17,18,19,20]. In fact, this disease occurs frequently in indigenous populations of the Americas that inhabit these kinds of environments [1,3,4,16,21,22]. Since CDK affects predominantly individuals in remote and impoverished marginal rural areas, identifying risk factors more accurately may give insights into ways to prevent it.

These observations suggested that susceptibility to CDK could be influenced by the genetic background of the individuals who exhibit the disease, in particular, that of Native Americans. For example, certain mtDNA-based eye diseases such as Leber’s Hereditary Optic Neuropathy (LHON) have exhibited population-based effects. This disorder has been observed more frequently in haplogroup J in Caucasian and Slavic populations than in other maternal lineages present in these populations [23,24,25,26], whereas it appears in East Eurasian lineages such as D that are more common in East Asian populations [27,28]. Likewise, the sequence composition of the mtDNA genome affects the expression of retinitis pigmentosa in adult onset neuropathy, ataxia and retinitis pigmentosa (NARP) cases caused by the T8993G ATPase 6 mutation [29]. Based on these data, there could be similar population genetic effects on the type and incidence of eye diseases that occur in Native Americans, such as CDK.

Another reason we suspected the involvement of mitochondria in CDK is their established role in other corneal diseases. For example, although corneal clouding occurs infrequently in infants and children, it is often associated with mucopolysaccharidoses or Fabry disease when it appears in these young individuals. Some of those cases have also exhibited abnormal mitochondria in muscle biopsies analyzed under light and electron microscopy, implying significant mitochondrial dysfunction [30]. Similarly, previous work on keratocornus (KC), a noninflammatory thinning disorder usually involving the central or inferior cornea, revealed that KC corneas exhibited more mtDNA damage than did normal corneas [31], suggesting that increased oxidative stress and compromised mtDNA integrity may be involved in KC pathogenesis [32]. Likewise, in certain cases of chronic progressive external ophthalmoplegia (CPEO), a mtDNA-based genetic disorder characterized by progressive ocular dysmotility, eyelid ptosis, optic atrophy and retinal pigmentary changes, bilateral segmental corneal oedema also occurs [33]. This kind of corneal damage has also been noted in other mtDNA based diseases such as Kearns–Sayre syndrome and ophthalmoplegia-plus [34]. Moreover, the autosomal dominant eye disease Schnyder corneal dystrophy (SCD), in which the abnormal deposition of cholesterol and phospholipids in the cornea results in progressive corneal opacification and visual loss, was linked to mutations in the UB1AD1 gene, which has been co-localized with a subunit of NADH dehydrogenase, a key complex I OXPHOS enzyme in mitochondria [35]. Thus, mitochondrial bioenergetics clearly plays a significant role in a variety of ophthalmological diseases, including those preferentially affecting the cornea.

In an initial effort to investigate the influence of genetic ancestry on the expression of CDK in indigenous populations of the Americas, and to begin exploring the possible role of mitochondrial bioenergetics in CDK expression, we examined mtDNA variation in 56 persons of Mapuche descent from the northwest area of Patagonia in Argentina. These individuals represented both CDK cases and controls. In addition, to assess the possible influence of non-native admixture on the incidence of CDK, we characterized Y-chromosome variation in males from the CDK and control populations. Through this analysis, we were able to characterize the maternal and paternal haplogroup diversity in these individuals, and assess the relationship between their genetic ancestry and disease status.

## Subjects and Methods

### Subjects

The participants in this study were mostly persons of Mapuche descent living in settlements and villages located at 40° S in the northwest Argentine Patagonia (Province of Rio Negro). Based on the clinical assessment of CDK, individuals were asked to participate in this study. In addition, persons not exhibiting signs of CDK but who were of similar age, sex and ethnic ancestry were also asked to participate and serve as controls for the CDK-positive individuals. CDK patients and controls did not have any other eye diseases, such as dry eye syndrome or rheumatoid arthritis, and were not on systemic medications at the time of this study. Together, they comprised a total of 56 individuals. Because this disease preferentially affects men, most of the participants were adult males.

### Ophthalmological examination and sample collection

Field research and sample collection were undertaken in this northwest Patagonia region in Argentina. The Argentine team conducted a complete eye examination as previously described [4], administered necessary health assistance, and took blood samples from persons living in communities within the region for genetic analyses, all under informed consent. In addition to providing all study participants with medical care and eye examinations, we also gathered demographic and genealogical information from them to obtain a better understanding of their genetic ancestry.

The protocol used in this work and written informed consent were approved by the Institutional Review Board of the Catholic University of Cordoba, the Institutional Committee of Ethics in Health Research, Ministry of Health of the Province of Cordoba, Argentina, and the Institutional Review Board #8 of the University of Pennsylvania. The study was conducted in accordance with the tenets of the Declaration of Helsinki. All participants provide their written informed consent to participate in this study.

### Molecular Genetic Methods

#### Mitochondrial DNA Analysis

The DNA samples were examined for mtDNA sequence variation through single nucleotide polymorphisms (SNP) analysis. The SNP analysis involved screening the samples for markers forming the basal portion of the mtDNA phylogeny (M, N, and R) and the diagnostic markers of East Eurasian (A-D) [36,37,38] haplogroups (maternal lineages) known to be present in Native American populations and haplogroups common in European groups [39,40]. The basal SNPs were assessed using custom TaqMan^®^ assays (Applied Biosystems, Bedford, MA, USA) and run on an ABI 7900HT Fast Real-Time PCR System. All other SNPs (A-D, X) were detected through PCR-RFLP analysis [41,42]. The SNP data were used to assign a haplogroup to each mtDNA. We also sequenced the entire mtDNA control region to ascertain individual haplotypes, using published methods [43,44,45].

#### Y-chromosome Analysis

Y-chromosome analysis utilized the informative SNPs identified in previous studies of world populations to characterize paternal haplotypes. The SNPs examined include M3, M9, M20, M45, M69, M89, M168, M170 and M201 [46,47,48], M242 [49,50], LLY22g [51], and M304 [52]. They were detected through Taq Man assays with fluorescent primers using the ABI 7900HT Fast Real-Time PCR System. Five markers (M60, M91, M139, M175 and M186) [48] were detected using a multiplex reaction, and read on the ABI 3130XL Gene Analyzer, using GeneMapper software. Paternal haplotypes were defined through the analysis of STR variation, using the ABI AmpFℓSTR® Y-Filer kit, following published methods [43,44,45].

## Results

We evaluated the results of the genetic analysis of the CDK and control samples in the context of the genealogical data from participants. This process allowed us to remove certain individuals from the statistical analysis because of consanguinity. For example, we noted that two control individuals were maternally related, and excluded one from further analysis. In addition, several other persons were maternally related, with three of them being controls and the other a CDK patient. We decided to keep one of the controls and the related CDK patient in the analysis, but included no other relatives in the statistical analysis. In addition, two of these individuals were paternally related, and, thus, one was excluded from the NRY analysis. All other individuals appeared to represent distinct lineages within the CDK and control groups. This evaluation reduced the CDK and control groups to 29 and 24 individuals, respectively for the mtDNA analysis, and for the NRY analysis, 26 and 18, respectively.

### mtDNA Results

In general, the mtDNA diversity observed in the Mapuche CDK patient and control samples were typical of those already described in South American populations [36,53,54,55,56]. With few exceptions, these individuals had mtDNAs from haplogroups commonly seen in Native Americans (Table 1). The majority of these mtDNAs belonged to haplogroup D (48%), with slightly over half of these haplotypes appearing in CDK patients. Haplogroup B was the next most frequent haplogroup, and appeared at a greater frequency (28%) than has been described previously in Mapuche groups [53,54]. Again, more than half of the B haplotypes appeared in CDK patients. Likewise, haplogroup C was nearly as common as haplogroup B in this population (23%), and occurred at a higher frequency than observed in other Mapuche groups [53,54]. However, only 5 of 12 haplogroup C individuals were CDK patients. Haplogroups A, K, and X were represented by single individuals, with the only CDK patient belonging to haplogroup K.

**Table 1:** mtDNA haplogroup frequencies of Mapuche CDK patients and controls.

**Haplogroup** *	**CDK**	**Control**
A	0 (0.000)	1 (0.042)
B	9 (0.310)	6 (0.250)
C	5 (0.172)	7 (0.292)
D	14 (0.483)	9 (0.375)
K	1 (0.034)	0 (0.000)
X	0 (0.000)	1 (0.042)
Total	29	24

(*) Haplogroups classified as indigenous (A, B, C, D) or non-indigenous (K, X) in origin.

Based on a Fisher’s Exact test (p = 0.705), there was no significant difference in the frequencies of mtDNA haplogroups between the CDK patient and control samples. In other words, there appeared to be no association between the incidence of CDK and the presence of an indigenous mtDNA haplogroup.

### Y-chromosome Results

Roughly half of the 44 Y-chromosomes belonged to haplogroup Q1a3a, which is commonly found throughout the Americas [50,57,58,59,60,61] (Table 2). The remaining Y-chromosomes belonged to haplogroups I, J, K and R. With the exception of haplogroup K, these paternal haplotypes are typically found in European populations [46,47,48], and, as such, can be considered the result of intermarriage between members of indigenous and colonizing populations. Most of the CDK patients had either haplogroup Q1a3a or R haplotypes, while the control individuals had a significantly higher frequency of non-indigenous Y-chromosome haplotypes than the patient group.

**Table 2:** Y-chromosome haplogroup frequencies of Mapuche CDK patients and controls.

**Haplogroup** *	**CDK**	**Controls**
I	1 (0.039)	2 (0.111)
J	1 (0.039)	0 (0.000)
K (x L, M1, NO, P)	0 (0.000)	3 (0.167)
Q1a3	1 (0.039)	0 (0.000)
Q1a3a	14 (0.539)	7 (0.389)
R	9 (0.346)	6 (0.333)
Total	26	18

(*) Haplogroups classified as indigenous (Q1a3, Q1a3a) or non-indigenous (I, J, K, R) in origin.

However, when statistically tested, these haplogroup differences were not significantly different (χ^2^ = 6.606, p =0.158). Similarly, there was no statistically significant relationship between an individual’s paternal genetic background and the incidence or stage of the disease (χ^2^ = 1.504, p = 0.220).

## Discussion

In this analysis, we investigated the relationship between the genetic background of CDK patient and control individuals of Mapuche descent and their disease status. In spite of the fact that the mtDNA plays a role in different kinds of eye diseases, there was no clear correlation between any particular maternal haplogroup and CDK status. Both CDK patients and controls had similar frequencies of the mtDNA lineages typically seen in Native American populations. In fact, we noted that, among the four individuals who were maternally related (a brother and sister and her two sons), only one of them had CDK. Thus, on this basis, CDK seems to be more strongly linked to environmental exposures, and not directly related to energetic deficiencies due to mtDNA mutations, as seen in LHON. On the other hand, given that the brother was the person with CDK, it is possible that his two nephews had not had sufficient time or exposure to develop the disease yet.

In addition, the difference in Y-chromosome haplogroup frequencies between the CDK and patient groups was intriguing. The degree of admixture in the control group, as measured by the presence of West Eurasian haplogroups, was considerably higher than in the CDK group, suggesting a potential paternal influence on disease status. Interestingly, previous work revealed similar results with the ABO system, with 64.5% of CDK patients and 44.8% of the controls being O/Rh+, respectively (Serra et al., unpublished data). However, as shown above, there was no statistically significant relationship between an individual’s paternal genetic background and the incidence or stage of the disease.

Therefore, these results indicate a lack of correlation between genetic ancestry as represented by these haploid genetic systems and the incidence of CDK in Mapuche populations.

In addition, the mtDNA appears plays less of a role in CDK expression than in complex diseases involving bioenergetic processes or other mitochondrial diseases affecting the eye. However, it should be noted that we have not yet examined the full mtDNA genome sequences of the CDK and control groups for putative pathogenic mutations, nor assessed the extent of DNA damage in corneal tissue obtained from transplant patients. Nevertheless, should there be genetic influence on the expression of CDK, our results point to nuclear genes being involved in disease etiology. The genetic loci possibly involved in the disease could include those involved in cell junction, focal adhesion and regulation of cytoskeleton, as indicated by recent proteomics work [14], those involved in corneal physiology, such as ALDH3A1 [62], or perhaps the UB1AD1 gene described above [34].

From a demographic point of view, it could be possible that we had an insufficient sample size to fully assess the association between genetic markers and CDK incidence. In this regard, our research group has been conducting ophthalmologic studies in individuals living in a vast, remote Patagonia region of Argentina (11,281 km^2^) for over eight years. Since the total population of this region is 2,329 inhabitants, giving a population density of 0.2 inhabitants per km^2^, the estimated size of the study sample should be 71 individuals. In this regard, we examined 758 individuals and found that 73 of them (9.6%) had CDK (85% male and 15% female). Upon deciding to investigate whether the incidence of and susceptibility to CDK was influenced by the genetic background of the individuals who exhibited the disorder, many of the patients had died, while others did not show up for examination and some decided not to participate in the study. For these reasons, we worked with as many CDK patients as were available and investigated a similar number of individuals without the disease who were randomly selected from the whole group. The data reported from the 56 participants therefore reflect the constraints of conducting population and medical genetics research with indigenous communities living in a vast and remote region of the country.
